# Climate change-related knowledge and attitudes among a sample of the general population in Egypt

**DOI:** 10.3389/fpubh.2022.1047301

**Published:** 2022-11-03

**Authors:** Marwa Rashad Salem, Nelly Hegazy, Anas Abdullnasser Thabet Mohammed, Esraa Mahrous Hassan, Mohamed Mohyeldin Saad Abdou, Marwa M. Zein

**Affiliations:** ^1^Department of Public Health and Community Medicine, Faculty of Medicine, Cairo University, Manial, Cairo, Egypt; ^2^Department of Public Health and Community Medicine, Faculty of Medicine, Helwan University, Helwan, Egypt; ^3^Faculty of Medicine, Cairo University, Cairo, Egypt; ^4^Faculty of Medicine, El Menia University, Minya, Egypt; ^5^Faculty of Medicine, Emergency Resident, Cairo University, Cairo, Egypt

**Keywords:** climate change, general population, Egypt, knowledge, attitudes

## Abstract

**Introduction:**

Identifying the public awareness and risk perception regarding climate change, are fundamental preliminary steps in determining gaps and paving the way for awareness campaigns that address climate change causes and counteraction mitigation measures. However, few studies were conducted in Egypt; thus, the researchers conducted the current cross-sectional study among a sample of the Egyptian population to identify general knowledge and perception about climate change and its effects, as well as attitudes toward mitigation measures.

**Methods:**

An exploratory population-based electronic-open survey, was conducted among 527 members of the general population between January and April 2022, using a convenience sampling technique. A pre-tested 2-page (screen) electronic included three sections: sociodemographic characteristics, global warming/climate change-related knowledge, and attitude toward climate change mitigation.

**Results:**

The average global warming knowledge score was 12 ± 3. More than 70% (71.1%) of the participants were knowledgeable (percentage score >70%). Approximately half of the enrolled participants (48.2%) agreed that everyone is vulnerable to the effects of global warming/climate change. More than three-quarters (78.3%) of the participants agreed that carbon emissions from vehicles and industrial methane emissions were the first factors that contributed to climate change, followed by the ozone holes (731%). Global warming/climate change-related knowledge was statistically higher in participants aged of >30 years, married participants, urban residents, highly educated individuals, and employed individuals (*p*-value ≤ 0.05). Approximately 80% of the participants agreed that responding to the questionnaire drew their attention to the topic of climate change and its effects. More than two-thirds of those polled agreed that increasing public transportation use could help mitigate the effects of climate change/global warming, followed by the materials used and the direction of construction.

**Conclusion:**

More than two-thirds of the participants were knowledgeable regarding climate change. Social media and the internet were the main sources of information. However, participants need to get the information in a different way that could help in changing their attitude positively toward the issue of climate change mitigation. The current study recommends the need for various initiatives that work should be launched.

## Introduction

Climate change (CC) is an unavoidable issue that poses a significant risk to human health on a global scale (1). Between 2030 and 2050, CC is expected to cause an additional 250,000 deaths per year due to starvation, malaria, diarrhea, and heat stress. By 2030, it is anticipated that direct health harm expenses will range from $2 to $4 billion annually (2). The areas least equipped to manage without support to plan and respond will be those with limited health infrastructure, which will largely be found in developing countries (2).

Despite its low contribution to greenhouse gas emissions, Africa is considered the most vulnerable continent to the impacts of CC (3). CC has resulted in rising temperatures, rising sea levels, changes in precipitation patterns, and more extreme weather events. All of these new changes in Africa endanger human health, food, and water security and impede socioeconomic development (4).

According to reports from the Intergovernmental Panel on Climate Change, Egypt, as a developing country and part of Africa, is recognized as highly vulnerable to CC impacts due to its geographical location and reliance on climate-sensitive economic sectors (5, 6). Egypt was ranked 107th out of 181 countries in the 2019 ND-GAIN Index, which informs the public sector about their vulnerabilities and readiness to deal with CC and its impacts and to prioritize their efforts and investments (7). Egypt is now facing waves of heavy rainfall, rising sea levels, and the risk of flooding coastal areas such as Alexandria, as well as a large area of the Nile Delta_ Egypt's most cultivated land_ which could have an adverse impact on national food security and result in economic loss (8). A study conducted in 2014, has anticipated that the continuing trend of decreasing agricultural production, malnutrition, hazardous health effects of particulate matter, and heat stress, combined with a subsequent decline in tourism revenues, will result in a substantial economic loss, estimated to reach 2–6%of Egypt's future gross domestic product (9).

Previous research has examined the perceptions of climate risks in either the developed or developing world to better understand the effects of CC on community health (10, 11). Another study revealed that public perceptions of CC and its impact on health may inform policies to deal with CC-related health challenges (12). A recent study conducted among the general population in Bangladesh revealed that the knowledge of CC was average and education was the most influential factor in understanding CC and its impact on health (13).

Identifying public awareness and risk perception regarding CC are critical preliminary steps in determining the gaps and paving the way for awareness campaigns that address CC causes, counteraction, and mitigation measures. However, few studies have been conducted in Egypt; thus, the researchers conducted the current exploratory cross-sectional study among a sample of the Egyptian populationto identify general knowledge and perception about CC and its effects, as well as attitudes toward mitigation measures. The study results can provide the foundation to develop community-based awareness campaigns, draw attention to the causes, indicators, and broad hazards of CC, and advocate for the urgent need to support strict governmental policies to combat it. Egypt has entered a golden age with the launch of the National Climate Change Strategy 2050, which prioritizes Egyptians' quality of life and seeks to serve as a road map to achieving the updated Egypt Vision 2030 (14).

## Materials and methods

### Study design

The current study is an exploratory population-based electronic-open survey that was conducted among a convenient sample of the general population during the study duration from January 2022 to April 2022. The research was presented following the Checklist for Reporting Results of Internet E Surveys (CHERRIES) guidelines (15).

### Sample size and sampling technique

The sample size was calculated using the following formula, *n* = required sample size, = 1.96, *P* = prevalence of the outcome (54%), based on a study conducted in Bangladesh in which the majority of the participants (54.2 %) had some knowledge of CC (13), E = margin of error; 0.05. Assuming a 25% non-response rate, a sample of 478 participants was required.

The inclusion criteria of participants were as follows: (i) being an Egyptian resident, (ii) being adults (≥18 years old), and (iii) and willing to participate.

### Data collection tools and techniques

A pre-tested 2-page (screen) electronic questionnaire was used to collect data from the study participants. It was divided into three sections:

1. Sociodemographic characteristics: age in years, gender, education, occupation (working or not), the field of study (biology, medicine, public health, others), marital status, and place of residence.
2. The knowledge of study participants regarding global warming and CC and related consequences was composed of 15 questions. The questions were formatted in closed-ended with yes, no, and do not know options.
3. Attitude toward efforts to combat CC: composed of six questions. The questions were formatted with close-ended agree, disagree, and neutral options.
4. Multiple options formats were used to obtain knowledge on factors contributing to CC and sources of knowledge about CC and global warming.

The questionnaire was adapted from previously published literature (16–20) (see Supplementary File I). Two language experts translated the questions into Arabic and then back-translated them into English by another two independent language experts.

Because of the COVID-19 critical situation to achieve social distance, the researchers used an online data collection method. A Google form was created, and participants were invited to fill it out and submit it. The researchers distributed the questionnaire link to groups on Facebook and WhatsApp. A pilot test was conducted with 10% of the calculated sample size (not included in the study) to assess the clarity of the questions. Two questions were deleted due to non-specific responses. The questionnaire's content was validated by four faculty members who are Public Health experts, and the necessary changes were made. Cronbach's alpha coefficient of 0.879 confirmed the reliability of the Knowledge and Risk Perception of CC and the global warming questionnaire.

### Statistical analysis

The researchers used SPSS (Statistical Package for Social Science) version 26.0 (IBM, SPSS, USA) for statistical analysis. Categorical variables were expressed as proportions and percentages. Quantitative variables were examined for normality. Quantitative variables were expressed using mean, standard deviation, median, and interquartile range (IQR); the researchers used the Chi-Square test of significance for comparison. A *P*-value of 0.05 or less was considered significant.

Knowledge question responses were coded 1 for yes responses and 0 for no and don't know responses. The 15 questions responses were added and the score percentage was calculated.

The knowledge percent score was grouped as knowledgeable ≥70%, not knowledgeable < 70% following Jamshidi et al., (21), and the individual scores were aggregated and grouped into five categories to indicate the overall knowledge level of respondents: not very knowledgeable (0–1), low knowledge (2–4), average Knowledge (5), knowledgeable (6–7), very knowledgeable (8–10) (22).

### Ethical considerations

The National Cancer Institute Cairo University Ethical Review Committee revised and approved the study protocol. The Ethical committee of the faculty of Medicine, Cairo University. All the included participants were treated according to the Helsinki Declaration of biomedical ethics. Before data collection, study participants electronically signed an informed consent after being informed about the purpose of the study and the significance of the online form. Participants were informed that the survey was anonymous and that participation was entirely voluntary. Data confidentiality was maintained throughout the study, and completed forms were accessed only by the investigators.

## Results

The questionnaire was opened 531 times and received 527 responses, with a response rate of 99.2%. The study participants ranged in age from 18 to 71 years old, with a mean age of 26 ± 8.

Approximately two thirds 65% of the participants were females, more than three-quarters (76.3%) were not married, and 60% of the enrolled participants lived in urban areas. The majority (82.5%) were highly educated, and more than two-thirds (66.2%) were not working. Most participants hadn't attended any training courses or workshops on CC or global warming during the past 12 months as displayed in Table 1.

**Table 1:** Baseline characteristics of the study participants (*N* = 527).

**Variables**	***N***	**%**
**Gender**		
Male	184	34.9
Female	343	65.1
**Marital status**		
Married	125	23.7
Not married	402	76.3
**Residence**		
Urban	316	60.0
Rural	211	40.0
**Education**		
Read and write	3	0.6
Secondary school	89	16.9
Higher education	435	82.5
**Occupation**		
Not Working	349	66.2
Working	178	33.8
**Is your study or occupation within the field of natural sciences (biology, medicine, public health)**		
Yes	319	60.5
No	208	39.5
**Have you attended any training courses or workshops on climate change or global warming and its effects during the past 12 months**		
Yes	30	5.7
No	497	94.3
**Have you heard of the terms global warming/climate change?**		
Yes	479	90.9
No	18	3.4
Don't know	30	5.7

The detailed correct answer to the 15 climate change and global warming knowledge questions was illustrated in Table 2. More than two thirds of the participants were knowledgeable about all the items concerned with the dangerous effects of climate change. The highest percentage was for increasing the possibility of extreme weather such as heat waves and extreme cold, ice melting, the incidence of floods, and that climate change will be more severe in the future. The least correct answer was for the following statement: Global worming increases the prevalence of malnutrition as reported by (61.2%) of the enrolled participants.

**Table 2:** Percent distribution of study participants by climate change/global warming knowledge.

**Knowledge questions**	**Yes**	**No**	**Don't know**
	***N* %**	***N* %**	***N* %**
Does global warming have an impact on human health	457 (95.4)	20 (4.2)	2 (0.4)
**Climate change**			
Increases the incidence of floods	390 (81.4)	75 (15.7)	14 (2.9)
Increases the water shortage problem	368 (76.8)	91 (19.0)	20 (4.2)
Is increasing the rate of glacier melting	445 (92.9)	24 (5.0)	10 (2.1)
Increases the possibility of extreme heat waves	454 (94.8)	18 (3.8)	7 (1.5)
Increases the likelihood of extreme cold	398 (83.1)	69 (14.4)	12 (2.5)
Increases the spread of diseases that are transmitted from one person to another such as gastroenteritis	352 (73.5)	102 (21.3)	25 (5.2)
Increases the prevalence of malnutrition diseases	293 (61.2)	163 (34.0)	23 (4.8)
Increases the likelihood of non-communicable diseases such as lung diseases such as asthma and respiratory problems	360 (75.2)	93 (19.4)	26 (5.4)
Affects mental health and increases anxiety and depression	337 (70.4)	125 (26.1)	17 (3.5)
Can impede health institutions to perform their role during severe cold spells or extreme heat	375 (78.3)	91 (19.0)	13 (2.7)
Displaces people and increases the number of refugees	354 (73.9)	112 (23.4)	13 (2.7)
Developed countries contribute more to climate change	344 (71.8)	123 (25.7)	12 (2.5)
Developing countries are more vulnerable to the effects of climate change	363 (75.8)	104 (21.7)	12 (2.5)
Climate change will be more severe in the future	410 (85.6)	50 (10.4)	19 (4.0)

**Figure 1:**
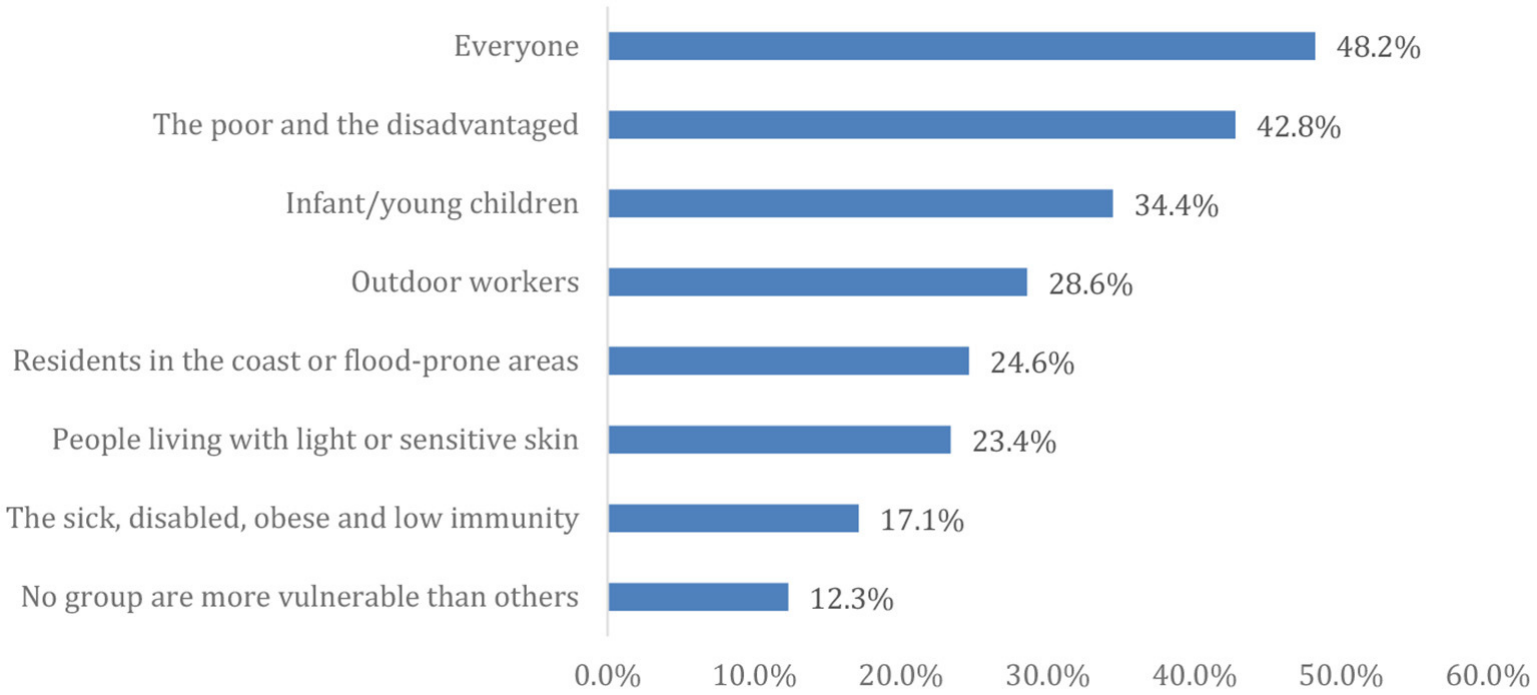
More vulnerable people to the effects of global warming/ climate change.

As shown in Figure 1, approximately half (48.2%) of the enrolled participants agreed that everyone is vulnerable to the effects of global warming/CC.

**Figure 2:**
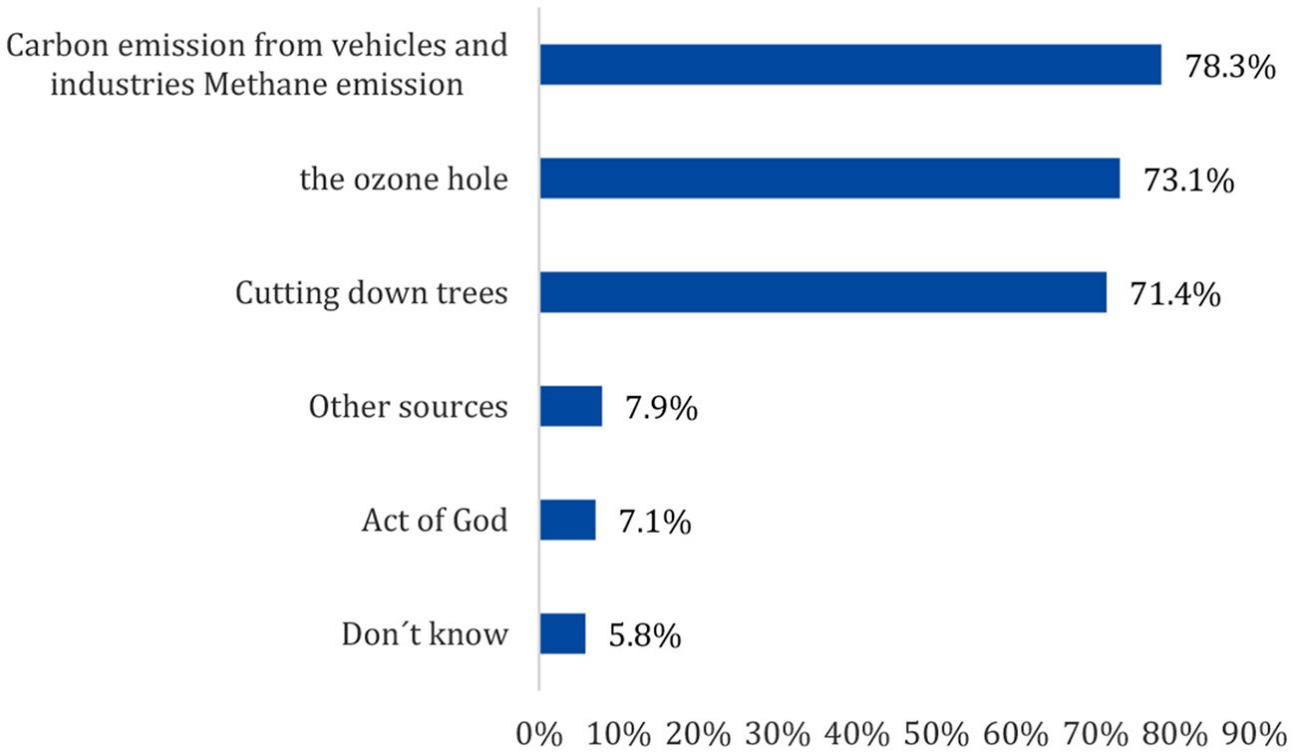
Factors contribute to climate change as reported by the enrolled participants.

Figure 2 depicts the factors contributing to CC; more than three-quarters (78.3%) of the participants agreed that carbon emissions from vehicles and industrial methane emissions were the first factors contributing to CC, followed by the ozone hole (73.1%).

Table 3 demonstrates the climate change/global warming knowledge score; the mean knowledge score was 12 ± 3, with a median score of 13 points (IQR 10, 14). The mean knowledge percent score was 79.5 ± 19.2, with a median of 86.7 (IQR 66.7, 93.3). More than three quarters (71.1%) of the participants were knowledgeable.

**Table 3:** Climate change/global warming knowledge score and knowledge percent score (*n* = 478).

Knowledge score mean ± sd, median (IQR)		12 ± 3	13 (10–14)
Knowledge percent score mean ± sd, median (IQR)		79.5± 19.2	86.7 (66.7–93.3)
Knowledge grouping	Knowledgeable (≥70%)	340 (71.1)	
	Not knowledgeable (< 70%)	138 (28.9)	
Level of knowledge	Not very knowledgeable (0%−20%)	5 (1.0)	
	Low knowledge (20%−40%)	13 (2.7)	
	Average knowledge (40%−60%)	35 (7.3)	
	Knowledgeable (60%−80%)	130 (27.2)	
	Very knowledgeable (80%−100%)	295 (61.7)	

**Figure 3:**
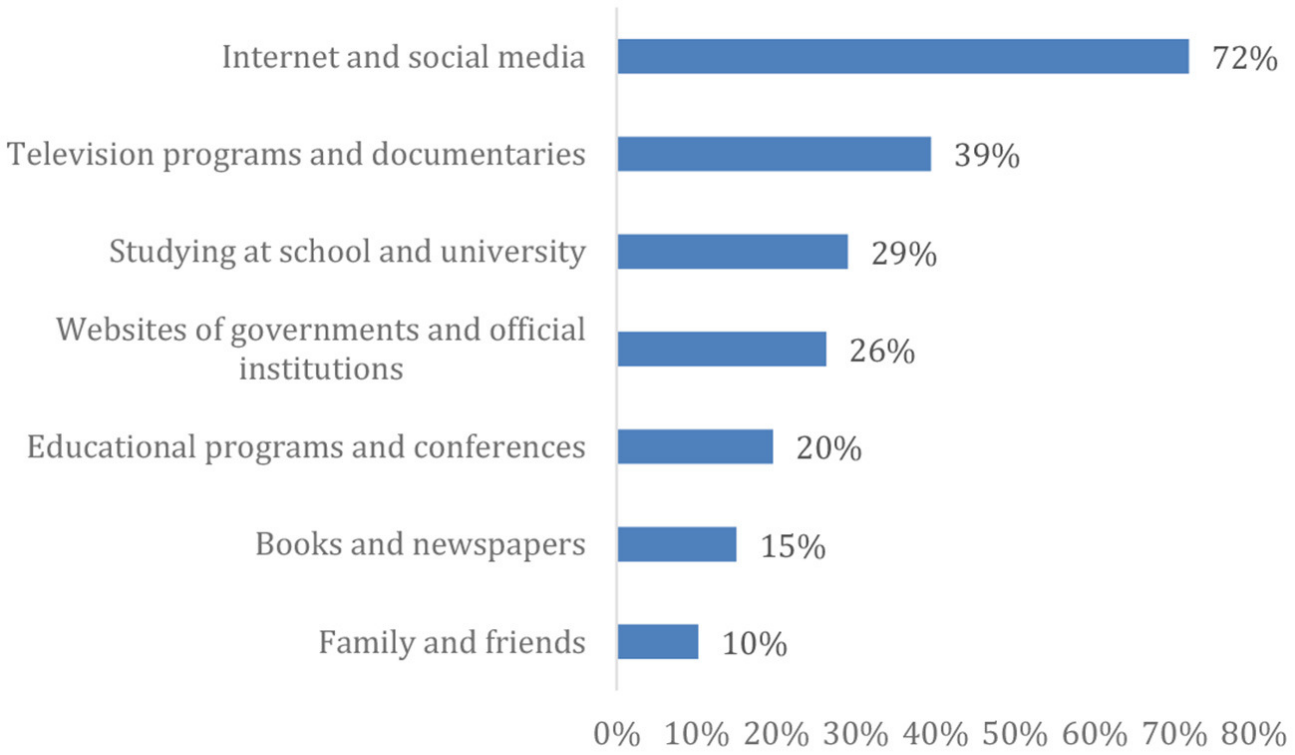
Percent distribution of the enrolled participants by source of knowledge.

As shown in Figure 3 more than 70% of the participants depend mainly on the internet and social media as primary sources of information on climate change/global warming.

As shown in Table 4, global warming/climate change-related knowledge was statistically higher in participants aged >30 years, married, urban residents, highly educated, and employed (*p*-value ≤ 0.05).

**Table 4:** Global warming/climate change level of knowledge with sociodemographic characteristics of the participants (*n* = 478).

**Variables**		**Knowledgeable**	**Not knowledgeable**	***P*-value**
		**(>70%)**	**(< 70%)**	
Age group	18–30	233 (65.8)	121 (34.2)	< 0.001^*^
	31–40	77 (88.5)	10 (11.5)	
	>40	30 (81.1)	7 (18.9)	
Gender	Male	119 (69.2)	53 (30.8)	0.482
	Female	221 (72.2)	85 (27.8)	
Marital status	Married	97 (81.5)	22 (18.5)	0.004^*^
	Not married	243 (67.7)	116 (32.3)	
Residence	Urban	237 (76.9)	71 (23.1)	< 0.001^*^
	Rural	103 (60.6)	67 (39.4)	
Education	Read and write	1 (100.0)	0 (0.0)	< 0.001^*^
	Secondary school	21 (38.9)	33 (61.1)	
	Higher education (university and postgraduates)	318 (75.2)	105 (24.8)	
Occupation	Not working	207 (67.2)	101 (32.8)	0.011^*^
	Working	133 (78.2)	37 (21.8)	
Your study or occupation within the field of natural sciences (biology, medicine, public health)	Yes	236 (76.1)	74 (23.9)	0.001^*^
	No	104 (61.9)	64 (38.1)	
Have you attended any training courses or workshops on climate change or global warming and its effects during the past 12 months	Yes	18 (64.3)	10 (35.7)	0.410
	No	322 (71.6)	128 (28.4)	

* Statistically significant.

**Figure 4:**
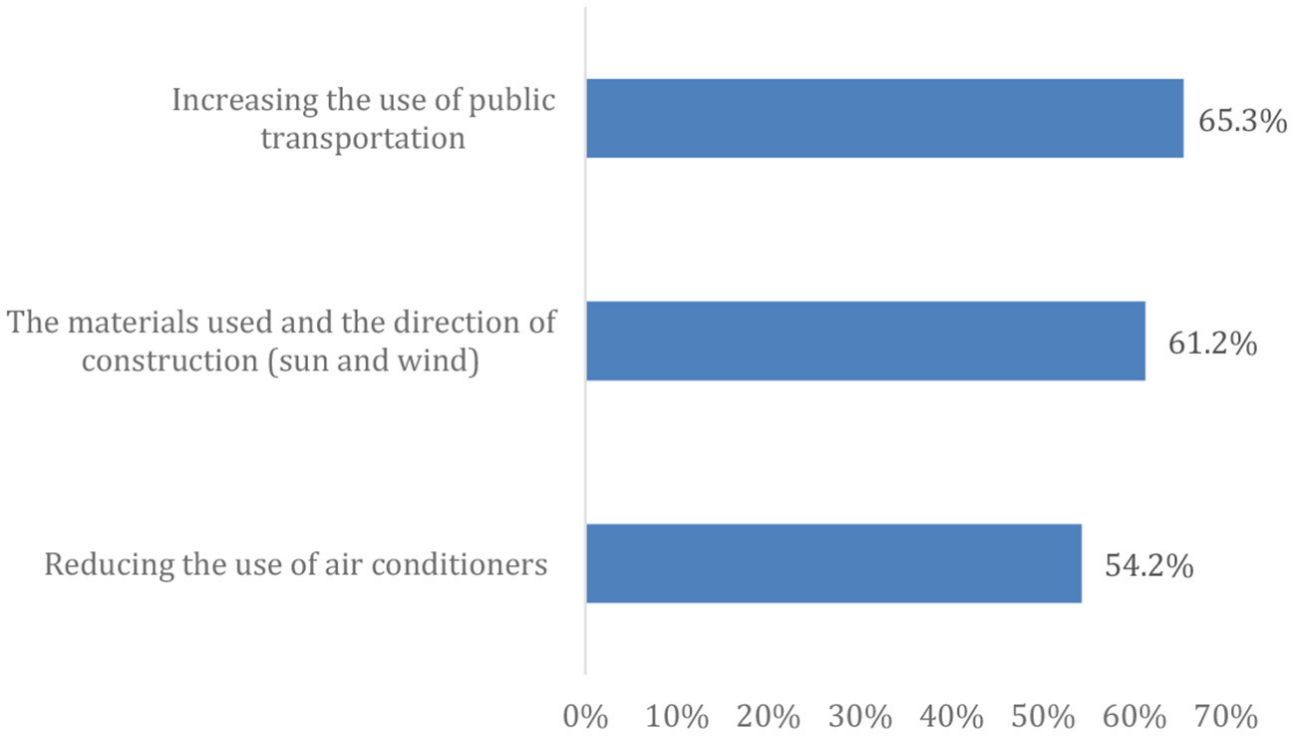
Positive attitude toward some factors that can contribute to reducing the impact of climate change.

Figure 4 depicts some mitigation measures, which revealed that more than two-thirds of the enrolled participants agreed that increasing public transportation use could reduce the effects of climate change/global warming, followed by the materials used and the construction direction.

**Figure 5:**
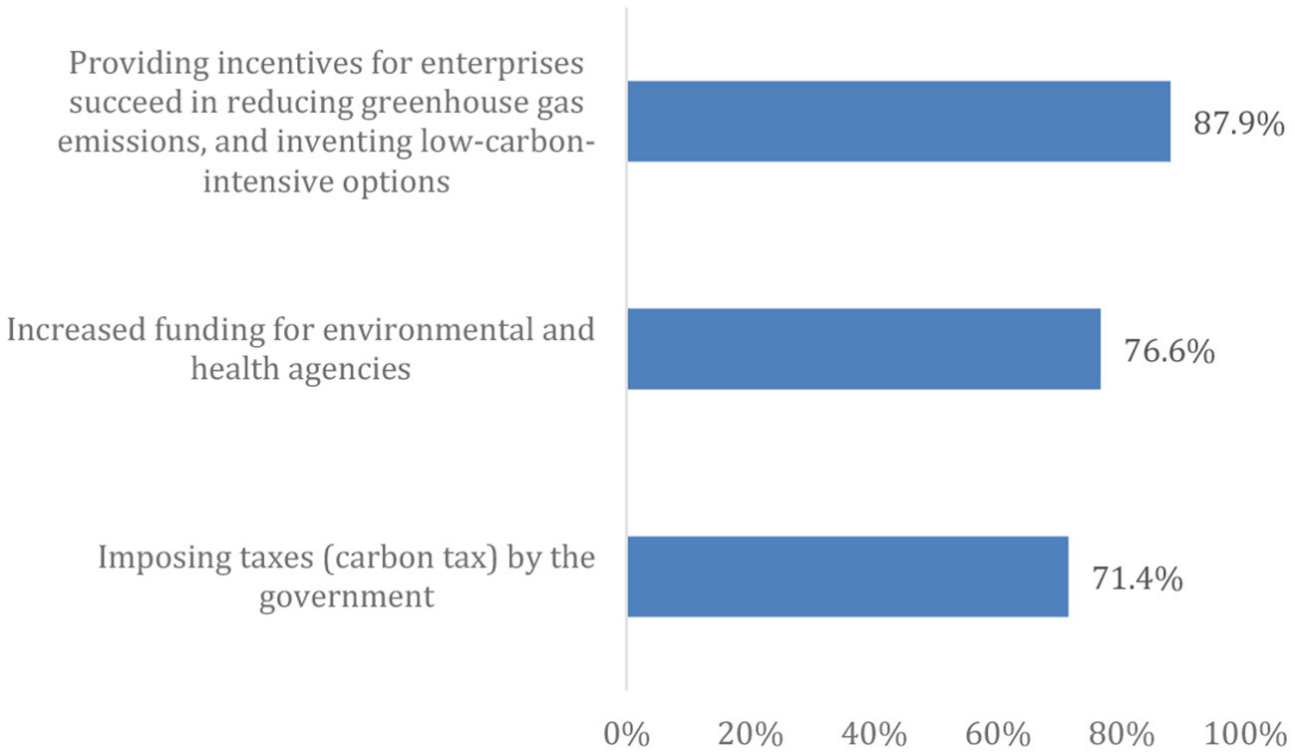
Positive attitude toward mitigation measures to reduce global warming.

Figure 5 illustrates that most of the respondents agreed on providing incentives to enterprises that succeed in reducing greenhouse gas emissions and inventing low carbon intensive options as a mitigation measure to combat global warming. Approximately 80% of participants agreed that answering the questionnaire drew their attention to the topic of CC and its effects, and more than 60% of the participants would like to learn more about it.

## Discussion

According to the current study's findings, 70% of the participants were knowledgeable about the issue of CC and its effects. These results were based on similar studies conducted in other countries, which revealed that more than 80% of the respondents were aware of the problem of CC and its consequences (17, 23). However, the research results contrast with studies conducted in Saudi Arabia, Turkey, and Kenya, which revealed a low level of knowledge about CC among participants (24–26). Notably, more than 75% of the participants were university graduates, with more than two-thirds of them having a field of study or occupation related to natural sciences, which have a significant association with high knowledge scores (27). These findings have shed light on the critical role of education in the issue of CC and the importance of incorporating it, as well as the urgent measures that should be implemented to mitigate its damaging consequences in the school and college curriculum (26–28).

More than two-thirds of the participants were well-versed in all aspects of CC's perilous consequences. The highest percentage predicted that extreme weather such as heat waves and extreme cold, ice melting, flood frequency, and CC will be more severe in the future (17). However, there is a gap in knowledge about who could be more affected by CC, which needs to be addressed. Less than half of the participants polled agreed that CC affects everyone, but certain groups could be more vulnerable than others (29).

Regarding the knowledge of the main causes of CC as an essential step in risk perception, approximately three-quarters of the participants chose carbon emissions from vehicles and industries, followed by the ozone hole, which increases the amounts of UV radiation reaching the Earth, and finally, deforestation. Only 7% of the voters chose an act of God. These findings are consistent with the results of studies conducted in Egypt, Saudi Arabia, and Oman (24, 30, 31). However, in a study conducted in Ghana, more than one-third of the respondents stated that deforestation is the primary cause of CC; interestingly, a small percentages of participants referred to carbon emissions as the cause of CC, with nearly similar votes given to an act of God (23).

Additionally, more than two-thirds of participants are optimistic about CC mitigation measures. Nearly two-thirds of participants supported the recommended mitigation measures, and the majority supported the idea of providing incentives to enterprises that succeed in reducing greenhouse gas emissions and developing low carbon-intensive alternatives. However, an important consideration should be noted while interpreting these findings that this study was conducted during the COVID-19 pandemic, which had a significant impact on the perceptions of CC. The detrimental social and economic disruption associated with the COVID-19 pandemic, as well as the significant loss of human life, have attracted people's attention to the interactions between human activities and the environment. Furthermore, pandemic control measures have included mobility restrictions and subsequent behavioral changes, such as working from home and reducing international travel, which consequently have been associated with temporary improvements in air quality and reductions in CO_2_ emissions in the environment (32). These circumstances have contributed to raising public awareness of the role of human activities in CC and encouraging a favorable attitude toward urgent policies to combat CC (33). Nonetheless, the public must be more motivated to protect their environment and work hard to restore it to its natural state and conserve its resources. This was evident in the results of a recent Egyptian study, in which climate change/global warming was ranked fifth regarding its significant impact on public life, and sixth regarding its importance to participants (28).

Different sources of information have various degrees of credibility, power of dissemination, and effect on the population. This plays a crucial role in raising public awareness regarding various issues and results in discrepancies in knowledge scores among di fferent populations. This study identified social media and the internet, followed by television programms and documentaries, as the primary sources of information for approximately three-quarters of the participants. Obtaining knowledge from schools or universities was prominent among only one-third of the participants (23). In a study conducted in Oman, TV channels, both international and local, were identified as the primary source, followed by school curricula, and lastly, social media and the internet (27). In a case study conducted in Egypt, most respondents stated that they trusted the information received mainly from scientists and environmental organizations, while approximately one-third relied on the media, such as TV and radio or governmental bodies (28).

The younger generation is our future. They will live longer and will be more vulnerable to the hazardous effects of CC. The next generation has the right to save the environment, preserve natural resources, and work on their efficient use. Consequently, climate literacy is a fundamental step. Consequently, cooperation with the ministry of education will be vital in integrating environmental topics into school and college curricula, considering their ages, to build a new generation highly aware of their environment, with strong values and attitudes, who are more knowledgeable about the required actions to save the environment, adapt to CC and be compatible with achieving the sustainable development goals and aligned with the updated Egypt Vision 2030 (33, 34).

## Conclusion

More than two-thirds of the participants were knowledgeable regarding CC. Social media and the internet were the main sources of information. However, participants need to get the information in a different way that could help in changing their attitude positively toward the issue of CC mitigation. The current study recommends the need for various initiatives that work in harmony to combat CC, such as educational campaigns, training workshops, and studies, which take advantage of the prominence of social media, the internet, and television channels for announcing and disseminating valid and credible information and promoting positive change in behavior.

### Study limitations

The current study findings should be viewed in light of the following limitations: The observational nature of the study. It was conducted to explore the situation in this new area of inquiry. It was not used to infer causal relationships. And due to the COVID-19 critical situation to achieve social distance, the researchers used the online data collection method. Consequently, the researchers do recommend conducting further studies using face to face interview using a probability sampling technique.

## Data availability statement

The raw data supporting the conclusions of this article will be made available by the authors, without undue reservation.

## Ethics statement

The studies involving human participants were reviewed and approved by National Cancer Institute. The patients/participants provided their written informed consent to participate in this study.

## Author contributions

MRS conceived the study, contributed to managing the literature searches, and data management. MMSA assisted with the literature search and writing. NH and MZ contributed to data analysis and results writing. AT and EM contributed to data collection and writing. All authors shared in data collection, drafting, approving the final manuscript in the study, contributed to the article, and approved the submitted version.

## Conflict of interest

The authors declare that the research was conducted in the absence of any commercial or financial relationships that could be construed as a potential conflict of interest.

## Publisher's note

All claims expressed in this article are solely those of the authors and do not necessarily represent those of their affiliated organizations, or those of the publisher, the editors and the reviewers. Any product that may be evaluated in this article, or claim that may be made by its manufacturer, is not guaranteed or endorsed by the publisher.
