# Supplementary material for: Climate change-related knowledge and attitudes among a sample of the general population in Egypt
Source: Front Public Health. 2022 Nov 3;10:1047301. doi: 10.3389/fpubh.2022.1047301 (PMC9669343; doi:10.3389/fpubh.2022.1047301)
Supplement: Supplementary file 1 [file Table_1.DOCX]

**Questionnaire to assess the knowledge of the general population on Global warming and climate change**

**Section I : sociodemographic data**

**Age: (years)**

**Gender:** male female

**Education:**

- Read and write
- Secondary school
- Higher education

**Marital status:**  Married Not married

**Working status:** Working Not working

**Is your study or occupation within the field of natural sciences (biology, medicine, public health):** Yes No

**Residence**: rural urban

**Section III: knowledge and Risk perception of climate change and global warming** :

Have you heard of the term global warming/climate change?

- Yes
- No
- I don’t know

|  |  | Yes | No | Don´t know |
| --- | --- | --- | --- | --- |
| 1 | Does global warming have an impact on human health |  |  |  |
| Climate change | |  |  |  |
| 2 | increases the incidence of floods |  |  |  |
| 3 | increases the water shortage problem |  |  |  |
| 4 | is increasing the rate of glacier melting |  |  |  |
| 5 | increases the possibility of extreme heat waves |  |  |  |
| 6 | increases the likelihood of extreme cold |  |  |  |
| 7 | increases the spread of diseases that are transmitted from one person to another such as gastroenteritis |  |  |  |
| 8 | increases the prevalence of malnutrition diseases |  |  |  |
| 9 | increases the likelihood of non-communicable diseases such as lung diseases, asthma, and respiratory problems |  |  |  |
| 10 | affects mental health and increases anxiety and depression |  |  |  |
| 11 | can impede health institutions to perform their role during severe cold spells or extreme heat |  |  |  |
| 12 | displaces people and increases the number of refugees |  |  |  |
| 13 | Developed countries contribute more to climate change |  |  |  |
| 14 | Developing countries are more vulnerable to the effects of climate change |  |  |  |
| 15 | Climate change will be more severe in the future |  |  |  |

**In your opinion, who is more vulnerable to the effects of global warming/ climate change? (you can choose more than one answer)**

- Infant/young children
- The poor and the disadvantaged
- Outdoor workers
- People living with light or sensitive skin
- Residents on the coast or in flood-prone areas
- The sick, disabled, obese, and with low immunity
- Everyone
- No group is more vulnerable than others

**Which of the following factors are likely to contribute to global warming and climate change? (you can choose more than one answer)**

- Carbon emission from vehicles and industries Methane emission
- the ozone hole
- Cutting down of trees
- Act of God
- others
- I don’t know

**Sources you get your information about global warming and climate change from (you can choose more than one answer)**

- Internet and social media
- Websites of governments and official institutions
- Television programs and documentaries
- Books and newspapers
- Family and friends
- Educational programs and conferences
- Studying at school and university

**Section III: Attitude towards efforts to combat climate change:**

|  | Attitude towards efforts to combat climate change: | Agree | Don’t agree | Neutral |
| --- | --- | --- | --- | --- |
| 1 | Do you think that reducing the use of air conditioners can contribute to reducing the effects of climate change? |  |  |  |
| 2 | Do you think that developing and increasing the use of public transportation can contribute to reducing the phenomenon of climate change |  |  |  |
| 3 | Do you think that taking into account the direction of construction (sun and wind) and the materials used have a decisive role in facing climate change? |  |  |  |
| 4 | Do you support the idea of providing incentives for enterprises that succeed in reducing greenhouse gas emissions, and inventing low-carbon-intensive options? |  |  |  |
| 5 | Do you support the government in imposing taxes that emitters pay for every ton of greenhouse gas emission (carbon tax) |  |  |  |
| 6 | Do you support that increased funding for environmental and health agencies would help protect against the health effects of climate change |  |  |  |

Did the previous questions draw your attention to the topic of climate change and its effects?

- Yes
- No

Would you like to know more about this topic?

- Yes
- No
